# Functional analysis of hot pepper ethylene responsive factor 1A in plant defense

**DOI:** 10.1080/15592324.2022.2027137

**Published:** 2022-02-22

**Authors:** Sung Un Huh

**Affiliations:** Department of Biology, Kunsan National University, Gunsan, Republic of Korea

**Keywords:** Ethylene-responsive factors (ERFs), hypersensitive response (HR) cell death, hot pepper, ethylene (ET), jasmonic acid (JA)

## Abstract

Ethylene-responsive factors play important roles in the biotic and abiotic stresses. Only some *ERF* genes from *Capsicum annuum* have been characterized. In the study, the *CaERF1A* gene is characterized in response to biotic stress. *CaERF1A* transcripts were induced by various plant defense-related hormone treatments. Knockdown of *CaERF1A* in hot pepper plants are negatively affected *Tobacco mosaic virus*-P_0_-mediated hypersensitive response cell death, resulting in reduced gene expression of pathogenesis-related genes and ethylene and jasmonic acid synthesis-related gene. Overexpressing *CaERF1A* transgenic plants show enhanced resistance to fungal pathogen via regulating ethylene and jasmonic acid synthesis-related gene expression. Thus, CaERF1A is a positive regulator of plant defense by modulating ethylene and jasmonic acid synthesis-related gene expressions.

## Introduction

Plants undergo many physiological changes to cope with various biotic challenges. The survival of plants mainly depends on their ability to evolve complicated defense system against different pathogen attacks through signaling networks^1,2^. In plants, transcription factors (TFs) play important roles in gene expression regulating response to biotic stress. Among the TF families, the apetala 2/ethylene-responsive factor (AP2/ERF) transcription factors are unique to plants and can be subdivided into four major subfamilies, AP2, related to abscisicacid insensitive 3/viviparous 1 (RAV), ethylene-responsive factor (ERF), and dehydration-responsive element-binding protein (DREB).^3–5^ AP2/ERF family is one of the largest TFs in the plant kingdom such as 122 *Arabidopsis*, 139 rice, 148 soybean, 121 barley, and 175 hot pepper genes.^3,6^ AP/ERF contains essential 68 amino acid repeat motifs that are designated the AP2 domain and contains DNA binding activity.^5^ ERF can activate or inhibit the transcription of genes that have GCC-box (AGCCGCC) motif in target gene promoters. Additionally, some ERFs also can bind to a CRT/DRE (A/GCCGAC) motif to regulate the expression of genes in response to biotic or abiotic stresses.^3^

Notably, ERFs are integrators of the hormonal pathways and directly responsible for the transcriptional regulation of several jasmonic acid (JA)/ethylene (ET)-responsive defense genes.^7,8^ Basically, constitutive expression of ERF family members known as positive activators of transcription is sufficient to activate the expression of JA/ET-dependent defense genes and to trigger resistance against necrotrophic pathogens.^9^ *Arabidopsis* ERF1 functions in the ET signaling pathway by activating the expression of *PDF1.2* and *PR3*.^10^ Constitutive expression of *AtERF1, AtERF6*, and *ORA59* was shown to enhance resistance against necrotrophic pathogens.^10–12^ Additionally, the ethylene-insensitive 3 (EIN3) and ethylene-insensitive 3 like-1 (EIL1) also play in the JA/ET signaling pathway and EIN3 directly bind to the ERF1 promoter to activate transcriptional activity.^7^ Expression of *ERF96* transcripts is highly upregulated after methyl jasmonate (MeJA) or the ET precursor 1-aminocyclopropane-1-carboxylic acid (ACC) treatment. *ERF96* overexpressing transgenic *Arabidopsis* plants show resistance phenotype to necrotrophic pathogens via increased up-regulation of JA/ET-dependent *PR* genes such as *PDF1.2, PR3*, and *PR4. NtERF5* overexpressing plants show a strong resistance to TMV infection.^13^ The role of ERFs in plant immunity is unlimited.

Here, CaERF1A is a positive regulator in biotic stress response against TMV and necrotrophic fungal pathogens. CaERF1A is required for full *L*-gene-mediated TMV resistance via regulation of ET/JA-synthesis-related gene expression and defense-related genes. This enhanced resistance is consistent with *A. alternata* infection in *CaERF1A* overexpressing *Arabidopsis* plants.

## Materials and methods

### Plant materials and growth conditions

Hot pepper (*Capsicum annuum* L.) cultivars Bugang and Nokkwang were grown in a growth room at 25°C with a 16 h light and 8 h dark photoperiod cycle. *Arabidopsis thaliana* Col-0 wild-type and transgenic plants were grown in a 16 h light/8 h dark photoperiod at 23°C in soil. For the constitutive expression of *CaERF1A* and *CaERF1A^nls^* mutant, a Gateway vector was used, and the *CaERF1A* open reading frame (ORF) was amplified using *Pfu*-polymerase and cloned. CaERF1A^nls^ construct was generated by deletion of putative nucleus localization signal (NLS) ‘KRRKK’ in the C-terminal using PCR.

### Virus-induced gene silencing (VIGS)

The 214 bp of 3’ untranslated region (UTR) and partial open reading frame (ORF) fragment of *CaERF1A* were cloned into the pTRV2 vector containing a part of the *Tobacco rattle virus* (TRV) genome using *Bam*HI sites. The *pTRV1* and *pTRV2-GFP* or *pTRV2-CaERF1A* were introduced into *Agrobacterium* strain GV3101. The Agro-cells are infiltrated using infiltration buffer (20 mM citric acid, 2% sucrose, and 200 μM acetosyringone, pH 5.2), adjusted to an OD_600_ = 0.2.

### Gene expression analysis

Six-week-old plants were used for virus inoculation and chemical treatments. Virus-containing sap TMV-P_0_ (avirulent) or PMMoV-P_1, 2, 3_ (virulent) strains were inoculated by rubbed fully expanded leaves with carborundum. For fugal pathogen inoculation, *Alternaria alternata* FBCC-F68 strain was grown and used for inoculation as described.^14^ In brief, *A. alternata* was grown on PDA plates for 6–8 d with 100% humidity at 25°C with a 16-h light/8-h dark cycle. *A. alternata* spore was inoculated by spray in the *Arabidopsis* leaves.

For chemical treatments, 6-week-old plants were sprayed with a solution of 10 mM salicylic acid (SA), 100 μM methyl jasmonate acid (MeJA), and 10 mM ethephon (ET). Total RNA was prepared with the samples using Trizol RNA extraction method. Quantitative RT-PCR was performed with SYBR Green according to the manufacturer’s instructions (KapaBiosystems). Primers used in these experiments are listed in Table S1.

### Enzyme-linked immunosorbent assay (ELISA) and Western blot

ELISA was performed as described previously.^15^ Total proteins were extracted from TMV-P_0_-inoculated plants. The polyclonal TMV-CP antibody (diluted 1:5000) was used, and plates were incubated for 1 h at 37°C. TBS buffer containing alkaline-phosphatase-conjugated goat anti-rabbit IgG was added and the plates were incubated further. *p*-Nitrophenyl phosphate was used as substrate and color values were measured at a wavelength of 405 nm by an ELISA reader. Western blot was performed with mCherry-specific antibody.

### Subcellular localization analysis of CaERF1A

*Arabidopsis* transgenic carrying *CaERF1A-mCherry* or *CaERF1A^nls^-mCherry* plant leaves were used for confocal microscope analysis. The mCherry signals were detected using an LSM 510 Meta confocal microscope (Carl Zeiss).

### Electrolyte leakage assay

The electrolyte leakage assay was conducted with leaves of hot pepper plants inoculated with TMV-P_0_.^16^ Briefly, five leaf discs were taken from one leaf and transferred to a six-well dish containing 5 ml water. After 1 hr of washing with gentle agitation, the leaf discs were transferred to new glass tubes containing 10 ml water. The conductivity of the samples was determined using portable conductivity meter (Thermo Orion). This experiment was repeated three times.

### Measurement of ethylene

The seedlings of *Arabidopsis CaERF1A*-OE transgenic and WT were grown in 500 mL uncapped air-tight plastic bottles (SPL) for 2 weeks at growth chamber. The *Arabidopsis* plants were inoculated with *A. alternata* FBCC-F68. Water was used as negative controls. After the bottles were sealed for the 24 hr, 1 mL of headspace of each bottle was measured for ethylene contents using gas chromatography with a flame ionization detector (Shimadzu).

## Results

### *CaERF1A*, a member of hot pepper AP/ERF family, is strongly induced by plant defense-related hormones and avirulent TMV-P_0_ inoculation

**Figure 1.**
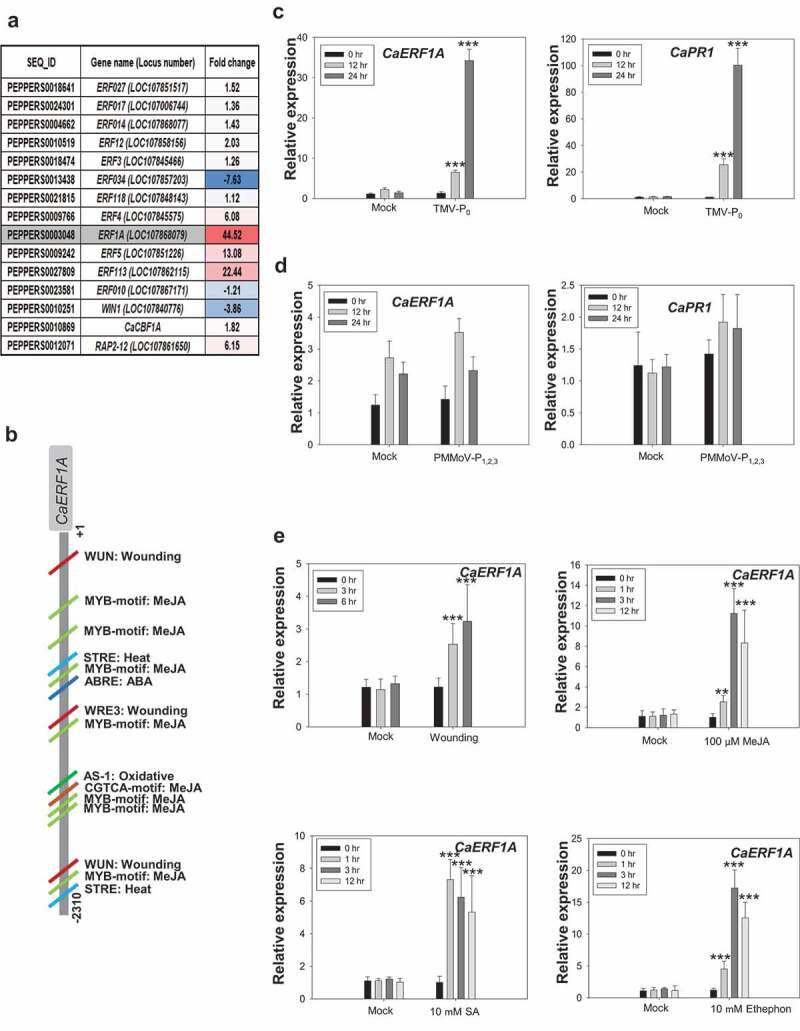
Expression analysis of *CaERF1A* under biotic stress condition. (a) Gene expression analysis of *Capsicum annuum ERF* transcription factors expression analysis using microarray data upon TMV infection.^17^ (b and c) Gene expression patterns of *CaERF1A* in avirulent strain TMV-P_0_-inoculated and virulent strain PMMoV-P_1,2,3_-inoculated hot pepper leaves. *CaPR1* was used as a positive control. Error bars indicate standard deviations (*n* = 3). Student’s *t*-test; ***P < 0.0001. (d) Schematic representation of the *CaERF1A promoter cis*-elements from hot pepper genome data. Each *cis*-acting regulatory elements (CAREs) were predicted by PlantCARE tool. (e) qRT-PCR analysis of *CaERF1A* in response to wounding, MeJA, SA, and ethylene. Error bars indicate standard deviations (*n* = 3). Student’s *t*-test; **P < 0 .001, ***P < 0.0001.

Previously, we performed microarray analysis of TMV-P_0_-mediated cell death condition to better understand the mechanism underlying *L*-gene-mediated cell death upon TMV infection.^17^ And we focused on the *ERF* genes that were up-regulated during TMV-P_0_ infection (Figure 1a). Among them, highly induced *CaERF1A* (LOC107868079) gene was selected. CaERF1A contains conserved AP/ERF DNA binding domain and has a putative ‘KRRKK’ nucleus localization signal peptide in the C-terminal region (Fig S1a). From the phylogenetic tress analysis, CaERF1A is close to group V *Arabidopsis* ERF1A and ERF2 but more closely related to tobacco ERF2 members (Fig. S1). To confirm the microarray data, we performed qRT-PCR analysis with TMV-P_0_ (avirulent strain) and PMMoV-P_1,2,3_ (virulent strain)-inoculated hot pepper leaves. Expression of *CaERF1A* is significantly increased in TMV-P_0_-inoculated plants but not PMMoV-P_1,2,3_, suggesting that CaERF1A is specifically involved in TMV-P_0_-mediated hypersensitive response but not basal defense (Figures 1b and 1c).

To further characterize the function of CaERF1A in plant defense, we analyzed the about 18 2 kb promoter region of *CaERF1A* using PlantCARE tool.^20^ In the analysis of the *CaERF1A* promoter, we found ERF1A function might be related to ethylene (ET)/jasmoic acid (JA)-mediated signaling (Figure 1d). Indeed, expression patterns of *CaERF1A* in hot pepper plants treated with wounding, methyl jasmonic acid (MeJA), ET, and salicylic acid (SA) were significantly increased (Figure 1e). These results indicate that CaERF1A function in the TMV-P_0_-mediated hypersensitive response cell death might regulate ET/JA-mediated signaling. Similarly, activation of AtERF1 requires 19 both ET and JA. *AtERF1* expression in JA-insensitive mutant is inactivated.^21^

**Figure 2.**
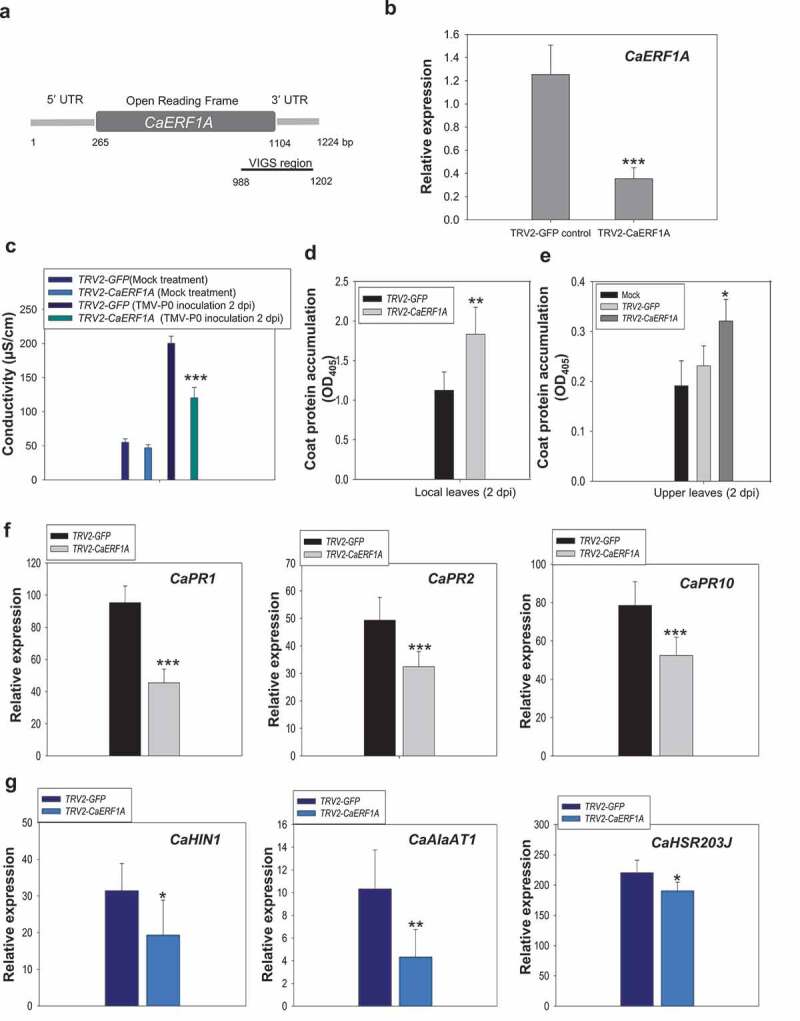
CaERF1A is involved in *L*-mediated resistance upon TMV-P_0_ infection. (a) A schematic illustration of VIGS region of *CaERF1A*. Partial 3’ UTR and ORF fragments of *CaERF1A* cDNA were used for VIGS. (b) Silencing efficiency of *CaERF1A* in *TRV2-CaERF1A*-silenced plants using qRT-PCR analysis. Error bars indicate standard deviations (*n* = 3). Student’s *t*-test; ***P < 0.0001. (c) For quantification of TMV-P_0_-mediated HR cell death, conductivity was measured by electrolyte leakage assay upon TMV-P_0_-inoculated or mock-treated *TRV2-CaERF1A* and *TRV2-GFP* plants at 3 dpi. The error bars show the mean of the standard deviation (SD) of the replicate samples. (d) For quantification of TMV-P_0_ accumulation, ELISA analysis was performed in TMV-P_0_-inoculated upper and local leaves at 3 dpi. (e) ELISA was performed in non-TMV-P_0_-inoculated upper leaves. The error bars show the mean value of the standard deviation (SD) of the replicate samples. The student’s *t*-test is used to check whether two sets of data differ significantly (*p < 0.01). (f and g) qRT-PCR analysis for basal defense-related genes (*CaPR1, CaPR2*, and *CaPR10*) and HR cell death-related genes (*CaHIN1, CaAlaAT1*, and *CaHSR203J*) in TMV-P_0_-inoculated *CaERF1A*-silenced plants at 2 dpi. The error bars show the mean value of the standard deviation (SD) of the replicate samples. The statistical significance of the difference was confirmed by Student’s *t*-test (*P < 0.01, **P < 0.001, ***P < 0.0001).

To examine whether CaERF1A functions in the TMV-P_0_-mediated hypersensitive response cell death, we generated a *CaERF1A*-silencing construct with *Tobacco rattle virus* (TRV)-based VIGS system (Figure 2a). Gene silencing efficiency of *CaERF1A* showed about 65% (Figure 2b). To test the effect of TMV-P_0_-mediated cell death in *TRV2-CaERF1A* and *TRV2-GFP*, electrolyte leakage was measured. The conductivity of *TRV2-CaERF1A* plants was dramatically reduced by about 52% compared with *TRV2-GFP* plants (Figure 2c). In addition, accumulations of TMV virus coat protein are significantly reduced local and upper leaves in *TRV2-CaERF1A* plants when compared with control *TRV2-GFP* plants (Figures 2d and 2e). Thus, CaERF1A could be involved in TMV-P_0_-mediated cell death.

### Knockdown of CaERF1A affects TMV-P_0_-mediated HR cell death and defense-related gene expression

To investigate the role of CaERF1A as a transcription factor, we also checked expression patterns of pathogenesis-related genes (*PRs*) and HR cell death-related genes in *TRV2-CaERF1A* plants upon TMV-P_0_ inoculation. As expected, gene expression levels of *CaRP1, CaPR2*, and *CaPR10* were significantly down-regulated in TMV-P_0_-inoculated *TRV2-CaERF1A* plants when compared with *TRV2-GFP* control (Figure 2f). Furthermore, *CaHIN1, CaAIaAT1*, and *CaHSR203L* transcript levels also reduced in TMV-P_0_-inoculated *TRV2-CaERF1A* plants when compared with *TRV2-GFP* plants (Figure 2g). Thus, our data indicated that CaERF1A has a transcriptional reprogramming function in the TMV-P_0_-mediated cell death.

### *CaERF1A* regulates ethylene and jasmonic synthesis-related gene expression upon TMV-P_0_-mediated HR response

**Figure 3.**
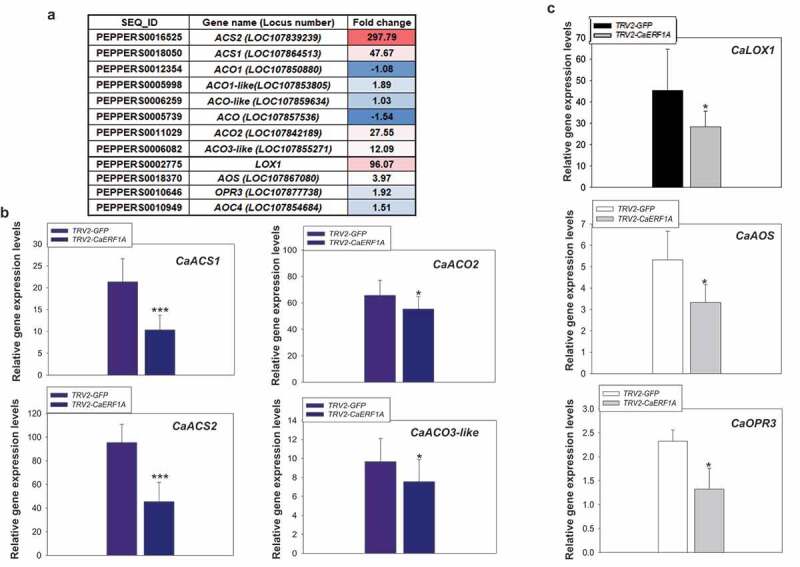
Suppression of ET/JA synthesis-related gene expression in TMV-P_0_-inoculated *CaERF1A*-silenced plants. (a) Gene expression analysis of ET/JA synthesis-related gene expression in TMV-P_0_-inoculated hot pepper plants using microarray data.^17^ (b and c) For monitoring of ET/JA synthesis-related genes expression patterns in TMV-P_0_-inoculated *CaERF1A*-silenced plants at 2 dpi, qRT-PCR analysis was performed using gene specific primers of ET synthesis-related genes (*CaACS1, CaACS2, CaACO2*, and *CaACO3-like*) and JA synthesis-related genes (*CaLOX*1, *CaAOS*, and *CaOPR3*). The statistical significance of the difference was confirmed by Student’s *t*-test (*P < 0.01, ***P < 0.0001).

JA, ET, and SA have shown that key elements for HR cell death and defense signaling control.^22–25^ To explore whether expression of ET/JA synthesis genes is changed in TMV-P_0_-mediated cell death, we analyzed expression patterns of ET/JA hormone synthesis gene in the microarray data. As expected, expression of some ET synthesis-related genes such as *1-aminocyclopropane-1-carboxylic acid* (*ACC), ACC synthase* (*ACS*), and *ACC oxidase* (*ACO*) was induced in TMV-P_0_-mediated cell death (Figure 3a). We also found that upregulation of JA synthesis-related genes such as *lipoxygense* (*LOX), allene oxide cyclase* (*AOC), allene oxide synthase* (*AOS*), and *12-oxophytodienoate reductase* (*OPR*) (Figure 3a), suggesting that TMV-P_0_-mediated cell death is required for ET/JA synthesis as well as SA. To confirm CaERF1A is involved in regulation of ET/JA synthesis-related gene expression in TMV-P_0_-mediated cell death, we performed qRT-PCR using *TRV2-CaERF1A* silencing and *TRV2-GFP* control plants upon TMV-P_0_ inoculation. Most of the ET/JA synthesis-related genes were reduced in TMV-P_0_-inoculated *TRV2-CaERF1A* silencing plants compared with *TRV2-GFP* plants (Figures 3b and 3c).

### *CaERF1A* overexpression in *Arabidopsis* enhances resistance to necrotrophic pathogen via ET and JA synthesis-related gene regulation

**Figure 4.**
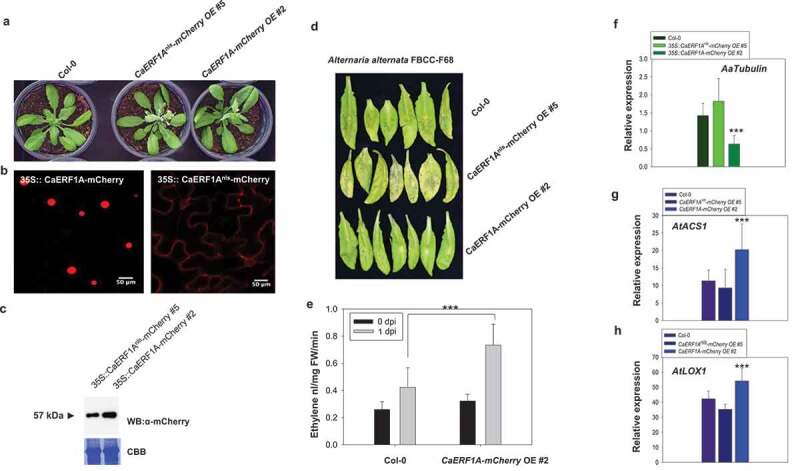
Overexpression of *CaERF1A* confers resistance to necrotrophic fungus in *Arabidopsis*. (a) *Arabidopsis* transgenic carrying *35::CaERF1A-mCherry* and NLS deletion mutant *35::CaERF1A^nls^-mCherry* plants shows no developmental phenotype. (b) CaERF1A localized to the nucleus but not CaERF1A^nls^ mutant in *Arabidopsis* transgenic plants. (c) Western blot analysis of CaERF1A-mCherry and CaERF1A^nls^-mCherry transgenic plants using mCherry specific antibody. (d) *Alternaria alternata* inoculation symptoms at 3 dpi. (e) The *Arabidopsis* transgenic carrying *35::CaERF1A-mCherry* plants showed increased ethylene production in response to *A. alternata*. After 1 dpi upon *A. alternata*, ethylene contents from seedlings of *35::CaERF1A-mCherry* transgenic and WT plants were measured using gas chromatography. The error bars show the mean value of the standard deviation of the replicate samples. The statistical significance of the difference was confirmed by Student’s *t*-test (***P < 0.0001). (f) *A. alternata Tubulin* gene expression analysis in the *A. alternata*-inoculated *35::CaERF1A-mCherry* transgenic and WT plants. The samples were harvested at 2 dpi. The error bars show the mean value of the standard deviation (SD) of the replicate samples. The statistical significance of the difference was confirmed by Student’s *t*-test (**P < 0.001). (g and h) Gene expression analysis of *AtACS1* and *AtLOX1*. The *35::CaERF1A-mCherry* transgenic and WT plants were inoculated by *A. alternata* and the samples were harvested at 2 dpi. The error bars show the mean value of the standard deviation (SD) of the replicate samples. The statistical significance of the difference was confirmed by Student’s *t*-test (***P < 0.0001).

To investigate gain of function of CaERF1A, we generated *35S::CaERF1A-mCherry* and *35S::CaERF1A^nls^-mCherry* transgenic *Arabidopsis* plants (Figure 4a). CaERF1A^nls^ construct was generated by deletion of putative nucleus localization signal (NLS) ‘KRRKK’ in the C-terminal end (Fig. S1). As expected, CaERF1A localizes in the nucleus and signal of CaERF1A^nls^-mCherry is mainly observed in the cytosol region but not nucleus (Figure 4b). We also conformed protein expression by Western blot in the *Arabidopsis* transgenic plants (Figure 4c).

We next tested whether CaERF1A might be required for ET/JA signaling-mediated resistance upon necrotrophic fungus, *Alternaria alternata* strain FBCC-F68. As shown Figure 4d, overexpression *CaERF1A* plants show more less symptoms when compared with control plants. Ethylene production was measured *A. alternate*-inoculated and non-inoculated *Arabidopsis* plants using gas chromatography. It was confirmed that ET produced more significantly in the *CaERF1A* transgenic plants compared with WT plant upon *A. alternate* inoculation (Figure 4e). We also checked accumulation of *A. alternata* with *A. alternata*-specific *Tubulin* gene primers. In *A. alternate*-inoculated *35S::CaERF1A-mCherry* transgenic plants, accumulations of *AaTubulin* gene are highly reduced when compared with control plants (Figure 4f). These results are consistent with reduced gene expression levels of *AtACS1* and *AtLOX1* in *A. alternate*-inoculated *35S::CaERF1A-mCherry* transgenic plants (Figures 4g and 4h). Thus, CaERF1A could enhance to JA/ET synthesis-related gene expression and ET production against fungal pathogen infection.

## Discussion

*Capsicum annuum* is an important horticultural crop that is susceptible to various pathogens, especially virus and necrotrophic fungal pathogens.^26,27^ Many transcription factors have been characterized for their defense role in hot pepper immunity to pathogens, but less information is known regarding the ERF family in hot pepper.

Ethylene-responsive factors (ERF) perform multifarious functions in plant resistance/tolerance to biotic and biotic stresses. The DNA-binding domain of AP2/ERF transcription factors, which consists of a sheet with three β-strands followed by α-helix, recognizes *cis*-regulatory elements, including the GCC- and GCC-boxes.^28,29^ Purified ethylene-responsive factor like protein 1 (CaERFLP1) protein was able to make a specific complex with both the GCC box and DRE/CRT motif.^30^ Overexpression of CaERFLP1 in transgenic tobacco plants showed improved resistance against the bacterial pathogen *Pseudomonas syringae*.^30^ Consistently, various defense-related genes, including GCC box-containing *PR* genes were constitutively expressed in *35S::CaERFLP1* tobacco plants.^30^ Identified ERFs regulated the expression of ET/JA-responsive genes by targeting GCC box present in their promoters.^31^ Similarly, CaERF1A also might bind to the GCC box of *ACS1* and *LOX1* promoters to regulate ET/JA synthesis against TMV and necrotic fungal pathogen infections.

The hot pepper plant contains CC-NB-LRR type *L*-gene resistance proteins, which confer resistance to *tobacco mosaic virus* (TMV) by restricting virus spread at the primary infection site.^32^ The resistance proteins are immune receptors that possess nucleotide-binding (NB) domain and leucine-rich repeat-containing (LRR) domains. They are part of a broad family conserved between plants and animals known as Nod-like receptors (NLR).^33^ NLRs mediate elicitor recognition and activate downstream signaling responses leading to programmed cell death termed the hypersensitive response.^34^ NLRs detect pathogen-derived effectors either directly or indirectly and then activate immune responses, including the accumulation of the defense hormones salicylic acid (SA) or jasmonic acid (JA), ethylene (ET), reactive oxygen species (ROS) and PR proteins.^35^ In *L*-gene-mediated resistance to TMV, we showed that expression of ET/JA biosynthesis-related genes are highly induced and ET production is induced upon fungal inoculation in *Arabidopsis 35S::CaERF1A* transgenic plants (Figure 4e).

SA is an important plant defense hormone, which positively regulates many ERFs.^36^ These data implied that JA/ET cross-talks with SA to fine-tune plants’ defense signaling pathway. Indeed, the transcript level of *CaERF1A* was strongly increased under JA/ET treatments. Thus, CaERF1A function might be associated with ET/JA signaling pathway in TMV-P_0_-mediated cell death. In *Arabidopsis*, ethylene signaling is required for the acceleration of cell death by activation mutant AtMEK5.^DD37 38 39^ Moreover, ethylene production is increased in TMV-inoculated *NN* tobacco plants and it is correlated with overexpression of *ACSs* and *ACOs*.^40,41^ Thus, CaERF1A is a positively regulator of ET/JA synthesis-related gene to enhance TMV-P_0_-mediated cell death and protect fungal pathogen infection.

## Conclusion

CaERF1A is identified with microarray analysis in TMV-P_0_-mediated cell death. AP/ERF transcription factors function as a positive or negative regulator in plant defense as well as environmental stresses. CaERF1A is a transcriptional positive regulator in the nucleus. CaERF1A is required for TMV-P_0_-mediated cell via regulating ET/JA synthesis-related gene expression. Furthermore, overexpression of *CaERF1A* confers a strong resistance against necrotrophic fungal pathogen infection. CaERF1A might be a useful genetic trait for engineering crop plant in the future.
